# Disease Transmission and Diagnosis of Zika Virus

**DOI:** 10.7759/cureus.49263

**Published:** 2023-11-22

**Authors:** Vinaya Bhandari, Avinash B Taksande, Bhagyesh Sapkale

**Affiliations:** 1 Medicine, Jawaharlal Nehru Medical College, Datta Meghe Institute of Higher Education and Research, Wardha, IND; 2 Physiology, Jawaharlal Nehru Medical College, Datta Meghe Institute of Higher Education and Research, Wardha, IND

**Keywords:** zika virus transmission, zika diagnosis, zika, zika virus, zika infection

## Abstract

An arbovirus belonging to the *Flaviviridae* family and the *Flavivirus* genus, the Zika virus (ZIKV), has profoundly transformed global health perception. Historically, ZIKV infections were considered infrequent, with generally mild manifestations. However, this perception changed dramatically when the virus quickly spread from Asia to the Americas, impacting many nations. It was alarming that there was a connection between ZIKV infection in pregnant women and the beginning of microcephaly in their offspring. ZIKV control and treatment are further complicated because *Aedes* mosquitoes, which primarily bite during the day, are the primary vectors of the virus. ZIKV diagnostic processes are complex since the virus shares symptoms with other illnesses like dengue and chikungunya. Despite the effectiveness of current diagnostic methods like real-time reverse transcription-polymerase chain reaction (RT-PCR) and enzyme-linked immunosorbent assay (ELISA), there is a clear need for more accurate antibody tests. This is especially true given that many people undergo testing while asymptomatic or after the ideal detection window. The capacity of ZIKV to infect human-derived neural progenitor cells raises worrying possibilities for severe neurological effects. With all these characteristics and their connection to birth abnormalities, research efforts into the virus's efficient treatment and prevention have increased. Overall, the emergence of ZIKV has demonstrated the necessity of a comprehensive and team-based strategy to address its myriad problems. This entails comprehending its transmission dynamics, enhancing diagnostic accuracy, and creating efficient therapies and preventive measures, all crucial to lessening the threat that ZIKV poses to the world's health.

## Introduction and background

The Zika virus (ZIKV), first identified in Africa in 1947, is a member of the genus *Flavivirus* and the family *Flaviviridae* which is transmitted by arachnids [1]. Due to outbreaks that included cases of severe symptoms like Guillain-Barré syndrome and microcephaly, it attracted attention from all around the world [2]. The history of the ZIKV outbreak from 2007 to 2019 is covered in the article, along with developments in vaccines and antiviral drugs, as well as genetics, transmission, and clinical symptoms [3]. Over 40 vaccine candidates are in preclinical research, and seven ZIKV-related products are currently on the market, thanks to structural investigations that identified commonalities with other flaviviruses [4,5]. When knowledge, funding, and cooperation are all in line, the quick creation of vaccines demonstrates efficient pandemic response capabilities. The crystal structure of the adult virus published in 2016 aided in creating vaccines [6]. The arbovirus ZIKV has a positive-sense, single-stranded RNA genome, and mosquitoes and different cell types are involved in its transmission cycle [7]. Real-time reverse transcription-polymerase chain reaction (RT-PCR) may identify the presence of viral genomes in fluids and can penetrate immune-privileged organs [8,9]. By breaking past the blood-tissue barrier, ZIKV spreads to immune-privileged organs like the brain, uterus, and testicles. To determine whether a person has a common ZIKV infection, RT-PCR can examine the viral genome in blood, saliva, urine, and other body fluids [9]. Bones, amniotic fluid, sperm, vaginal fluids, and breast secretions play crucial roles in understanding the transmission dynamics of the ZIKV. Using indirect techniques to detect ZIKV antibodies in blood, samples from milk and oropharyngeal secretions can be tested. The infectious disease research field is constantly changing, with discoveries, global influences influencing local environments, and public health organizations updating their guidelines. This makes a new narrative review on the ZIKV imperative. The review attempts to compile and summarize the most recent data to fill in the knowledge gaps from earlier studies and advance our understanding of the virus. Worldwide reports of ZIKV infection have come from all corners of the world and have transcended all national boundaries [10]. Furthermore, the necessity for a current and thorough overview is highlighted by its role in managing and avoiding future outbreaks and educating the public and healthcare professionals. Since it was essential to spreading awareness of the ZIKV globally, the article focuses on outbreaks prior to the 2007 Yap Island surge. Fifty years before to the Yap Island pandemic, ZIKV was known to sporadically occur [11]. The virus gained prominence after the 2007 epidemic, which was a major turning point in its history. Therefore, in order to fully comprehend the evolutionary patterns and historical trajectory of ZIKV, it is imperative that previous outbreaks be properly investigated. The essay limited the narrative review to the ZIKV and left out other viral infections in order to give a comprehensive and targeted analysis of the virus's unique characteristics, historical relevance, and complex public health issues. As a result, they were able to offer scholars, healthcare providers, and legislators a thorough resource.

## Review

Search methodology

A thorough and methodical search approach was used to gain a full understanding of the ZIKV and its effects on world health. Peer-reviewed papers, epidemiological research, and clinical reports on the ZIKV were mostly sought after using scientific databases, including PubMed, ScienceDirect, and Google Scholar. The search was restricted to publications from 1947 the year ZIKV was originally isolated until 2023 to locate the most recent results. Secondary terms such as "transmission", "diagnosis", "symptoms", and "treatment" were sometimes included in order to focus the search. Terms can be included or removed using the Boolean operators "AND" and "OR" as appropriate. For instance, searches like "Zika virus AND microcephaly", "ZIKV AND diagnosis", and "Flavivirus AND transmission" yielded specific results on the relationships between ZIKV and microcephaly, diagnostic methods for ZIKV, and transmission patterns of *Flavivirus*, respectively. Primary search terms included "Zika virus", "ZIKV", "Flaviviridae", and "Flavivirus".

**Figure 1:**
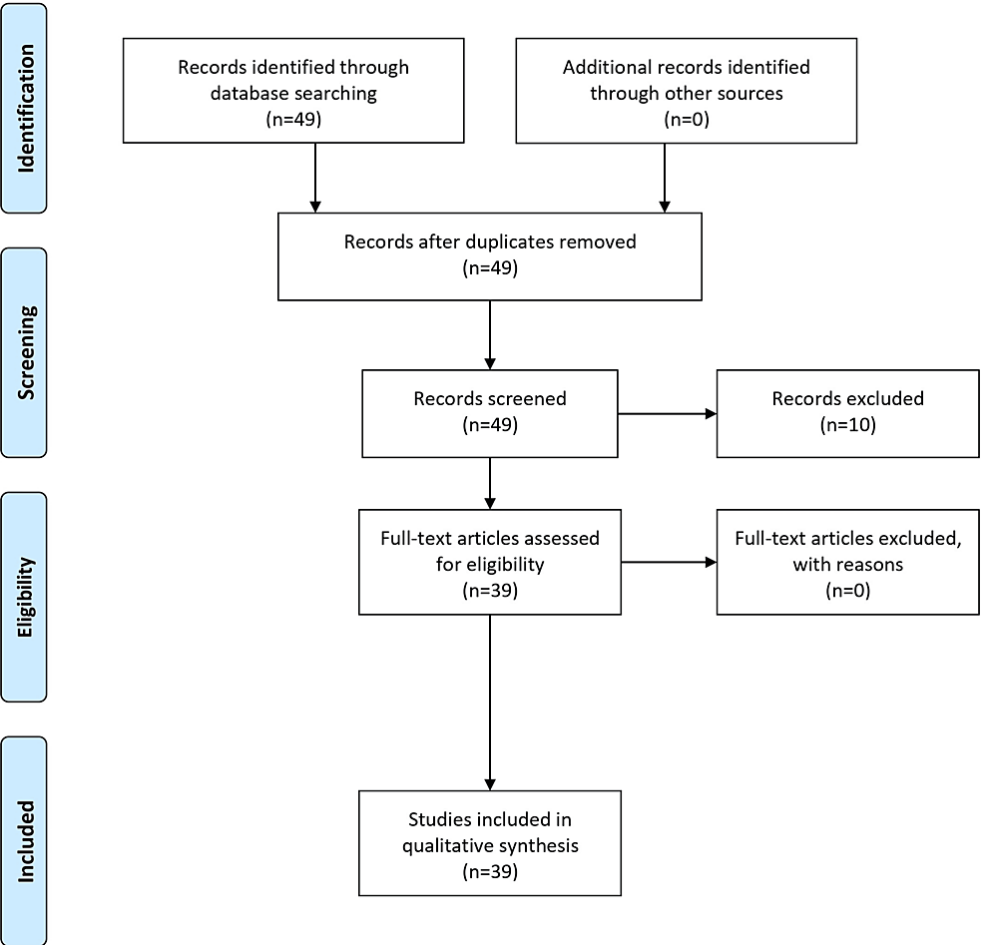
PRISMA flow diagram PRISMA: Preferred Reporting Items for Systematic Reviews and Meta-Analysis

Further, to understand the historical emergence and spread of ZIKV, specific filters were applied to retrieve articles focusing on outbreaks before the 2007 Yap Island surge and subsequent spread to the Americas. Special attention was also paid to articles detailing the molecular structure of the virus, its mechanism of transmission, and its potential link to neurological disorders. Given the similar symptomatology between ZIKV, dengue, and chikungunya, a comparative search strategy was also employed to identify these diseases' unique clinical presentations and diagnostic challenges. In conclusion, this search methodology was designed to offer a comprehensive and nuanced understanding of ZIKV, ensuring the retrieval of historical and the most recent scientific information. The Preferred Reporting Items for Systematic Reviews and Meta-Analysis (PRISMA) flow diagram is shown in Figure 1.

ZIKV

A developing *Flaviviridae* family and *Flavivirus* genus arbovirus known as ZIKV poses a danger to people's health [12]. Because ZIKV infections are infrequent and have modest symptoms, they have long been disregarded [13]. The ZIKV is an arthropod-borne virus that is a member of the *Flavivirus* genus in the *Flaviviridae* family. It shares strong ties with other members of the same family, including the viruses that cause Japanese encephalitis, dengue, yellow fever, and West Nile [14]. However, more recent data show a substantial correlation between ZIKV infection and microcephaly in children. More than 30 countries have been affected by the virus, which moved fast from Asia to the Americas in pregnant women. *Aedes* mosquitoes, which typically bite during the day, are the principal vectors of the ZIKV. Most persons contracting the ZIKV do not have any symptoms; however, those who do frequently experience two- to seven-day-long symptoms such as rash, fever, soreness in the muscles and joints, malaise, conjunctivitis, and headache [15]. It is an encapsulated virus with a positive-sense single-stranded ribonucleic acid (RNA) genome considered 10.7 Kb in size. The ZIKV genome encodes a unique open reading frame divided into seven nonstructural proteins from a polyprotein and three structural proteins post-translationally by viral proteases and host [16]. Serologic surveys suggest that although ZIKV-associated diseases may go unreported or be misdiagnosed, ZIKV infections can be rather common among people in southeast Senegal and other parts of Africa [17]. Much research has been done on the processes governing viral cell death. Viral-induced cellular stress and innate immune responses can cause apoptosis due to various viral determinants through various routes [18]. Infants with severe neurological conditions, birth deformities, and microcephaly have been affected by the ZIKV, which causes ZIKV disease in people. In an outbreak of vector-borne diseases, the vector population must have a high proportion of susceptible mosquitoes capable of capturing and transmitting the virus at a minimum infectious threshold [19]. Flaviviruses contain positive-sense single-stranded RNA genomes containing all the genes required for replication in mammalian and insect cells. The untranslated region (UTR) necessary for viral replication is the structural end of the genome at the 5' and 3' ends [20].

ZIKV and its history

ZIKV was first separated from a guard rhesus monkey in 1947 in Uganda, adjacent to the Zika Forest [21]. The Federated States of Micronesia's Yap Island experienced an outbreak in 2007 [11]. However, there is little proof that human infection existed earlier than that. ZIKV is a Spondweni group virus spread by mosquitoes that belongs to the *Flaviviridae* family and *Flavivirus* genus. There are two more genera in this family besides flaviviruses (genus *Flavivirus*): hepaciviruses and pestiviruses [22]. An outbreak that began in French Polynesia in October 2013 was caused by ZIKV [23]. Each of the three genera has a unique viral protein complement (and function) and minimal antigenic activity. Individual viruses within a genus encode homologous proteins with various degrees of antigenic similarity and similar activities. The frequency of strain GZ01 illnesses transmitted by mosquitoes is decreased by NS1 neutralization through antibody therapy [24]. Epidemiological, ecological, and virological studies have all been conducted. Still, current knowledge of the factors that led to the emergence of other *Aedes* virus diseases is incomplete. Like dengue and chikungunya, epidemiology or ecology predicts that virus genetic alterations will likely result in the formation of a virus strain with greater transmissibility, increased epidemic potential, and possibly toxicity [25]. ZIKV seroprevalence of up to 26% in asymptomatic African residents has been observed in Angola [26].

ZIKV transmission

By inducing pluripotent stem cells (hiPSCs), the ZIKV can infect human-derived neural progenitor cells in vitro, indicating that the virus may have the capacity to infect progenitor cells [27]. The 3' end of the viral RNA genome appears to have a higher prevalence of m6A methylation in the genomes of the flaviviruses under study. Furthermore, the dengue virus (DENV) and yellow fever virus (YFV) genomes' 3'UTRs and coding regions also experienced methylation [28]. No m6A was found in any of the two ZIKV genomes under study. However, m6A methylation increased at the 3' ends of the coding region. The potential for ZIKV sexual transmission highlights the need for more excellent information on the dynamics of virus persistence in semen in males residing in or travelling to places where ZIKV is spread [29]. With a mean lifespan of roughly 30 days, much longer than in serum or urine, detecting ZIKV RNA in semen has received extensive documentation [30]. Table 1 shows the modes of transmission of ZIKV and their related details.

**Table 1:** Modes of transmission of the Zika virus and their related details

Modes of transmission	Details
Mosquitoes	*Aedes* (*Stegomyia*) genus, mainly *Aedes aegypti*.
	Primarily in tropical and subtropical regions.
	*Aedes* mosquitoes typically bite during the day.
	Also responsible for transmitting dengue, chikungunya, and urban yellow fever.
Mother to fetus	Transmission occurs during pregnancy.
Sexual contact	It is transmitted between sexual partners.
Transfusion of blood and blood products	Risk of transmission through infected blood or blood products.
Organ transplantation	Potential transmission through transplantation of infected organs (though this mode is less documented).

Modes of Transmission

Mosquitoes, particularly those of the *Aedes* (*Stegomyia*) genus, most notably *Aedes aegypti*, are essential carriers of several diseases. Regions with tropical and subtropical climates frequently host these insects. *Aedes* mosquitoes are diurnal, which means they often bite during the daytime, unlike many other species. They are also in charge of spreading urban yellow fever in addition to dengue and chikungunya. Transmission during pregnancy can happen from the mother to the fetus in addition to mosquito bites. Additionally, partners' sexual interaction with one another might spread the illness. The disease can also spread by transferring contaminated blood and blood products. There is also a chance of transmission during organ transplantation if the organ comes from an infected donor, despite this possibility being less well documented. ZIKV transmission occurs through several notable methods. One of the main modes of transmission is through mosquitoes, especially those of the genus *Aedes* (*Stegomyia*), with the main culprit being *Aedes aegypti* [28]. These mosquitoes are mainly found in tropical and subtropical regions. What's remarkable about *Aedes* mosquitoes is their typical daytime biting habits.

In addition, it should be mentioned that these mosquitoes are not exclusive carriers of the ZIKV; they are also responsible for spreading other significant diseases, such as dengue, chikungunya, and urban yellow fever. Another important mode of ZIKV transmission is from the mother to the fetus during pregnancy. This vertical transmission has received particular attention because of the severe complications it can cause in infants, including microcephaly. Sex is another way for the ZIKV to be transmitted. The virus can be spread between sexual partners, underscoring the importance of protection and awareness even in areas where mosquito-borne diseases may not be typical. In addition, blood and blood products can be transfused to spread the ZIKV [29]. This has posed challenges for blood banks in areas where the virus is prevalent, requiring rigorous screening procedures. Finally, there is a potential, albeit less well-documented, risk associated with organ transplantation. Organs from infected donors can carry the virus, highlighting the need for thorough screening procedures before transplantation.

ZIKV diagnosis

It manifests as moderate fever, arthralgia, and occasionally arthritis. The second and third days of the fever are characterized by a variable, though potentially high, prevalence of conjunctivitis and a maculopapular rash in patients [31]. This does not last more than three to five days, and the rash will disappear shortly. A moderate amount of progress has been made with protease ZIKV. With a Ki of 361 ± 19 nM, the classic serine protease inhibitor aprotinin (BPTI) is a potent inhibitor of protein fusion [32]. Because of the larger vial volume and extended excretion period, whole blood and urine are also advised for testing in addition to serum [33]. Nucleic acid amplification testing, however, is not always helpful since many people are asymptomatic or show up for testing soon after ZIKV has been shed. Therefore, the most pressing requirement and most challenging task is to provide reliable antibody tests for the detection of recent ZIKV infection. ZIKV's nonstructural protein 5 (NS5) is an RNA-dependent RNA polymerase in charge of replicating the viral genome [34]. Notably, its structure was recently discovered, which will benefit the development of ZIKV antiviral drugs based on structure.

Most Common Symptoms of ZIKV Disease

Numerous ZIKV victims will not or only experience minor symptoms such as fever, rash, headache, arthritis, conjunctivitis (red eye), and muscle pain [35]. ZIKV is usually mild, with symptoms lasting from a few days to a week. For specimens that test negative for dengue and chikungunya, screening for the diagnosis of ZIKV is recommended. RT-PCR targeting the genomic area of nonstructural proteins is the primary method of diagnosis [36]. Despite limitations and accuracy concerns, point-of-care techniques such as immunochromatographic assays and serological tests that identify ZIKV antibodies play a critical role as alternative diagnostic approaches for ZIKV identification in resource-constrained areas when RT-PCR is unavailable. Even though viral RNA has been found in serum a maximum of 10 days after the beginning of symptoms and in other bodily fluids, including saliva or urine, more research is required to corroborate these results. With current knowledge, serum samples should be obtained within the first five days after the onset of symptoms. The ZIKV, which is spread by mosquitoes, exhibits various symptoms with varying degrees of seriousness. Mild fever is frequently one of the main signs of ZIKV infection. Symptoms of the ZIKV are shown in Table 2.

**Table 2:** Symptoms of the Zika virus Various symptoms of the Zika virus infection might vary in severity. A low temperature is frequently one of the first signs of an infection. People may notice a prominent, occasionally itchy skin rash that can spread to different body places along with the fever. Those who are afflicted frequently complain of mild to severe headaches. Another sign is joint discomfort, which mainly affects the hands and feet and can range in intensity. Additionally, persons who have the virus frequently report muscle soreness or discomfort. Some people may also get conjunctivitis, which causes their eyes to become red and even gritty. In addition to leakage, this eye ailment may occasionally accompany pain.

Symptoms of Zika virus	Description
Fever	Often mild, it's one of the primary indicators of the Zika virus infection.
Rash	A noticeable skin rash that may be itchy and spread across different parts of the body.
Headache	Frequent moderate to severe headaches are common among those infected.
Joint pain	The pain usually affects the hands and feet and can sometimes be severe.
Muscle pain	Muscular discomfort or pain is a frequently reported symptom.
Conjunctivitis (red eyes)	The eyes become red and may feel gritty, accompanied by discharge and sometimes pain.
Malaise	A general discomfort, illness, or lack of well-being can be experienced.

Along with this, there are other prominent signs, including a rash on the skin. This rash is noticeable and can spread to various body areas, making those affected itchy. Also, headaches, ranging in severity from moderate to severe, are common among ZIKV patients. Joint pain is also typical, particularly in the hands and feet. Sometimes, this pain can become very acute, making it difficult to do daily duties. Muscle soreness is another common sign of infection that causes difficulty with movement and daily activities. Conjunctivitis, often known as red eyes, is another distinguishing ZIKV symptom [35]. Due to its simultaneous onset with other acute symptoms, correlation with classical ZIKV manifestations, viral tropism targeting ocular tissues, prolonged duration, and particular geographical and temporal patterns, conjunctivitis in ZIKV infections is unique. As a result, it is a clinically relevant and distinguishing symptom [11,24]. People who experience this symptom will notice that their eyes are becoming red, frequently accompanied by a gritty sensation. An eye discharge may occasionally occur, and the disease can get uncomfortable. The last symptom of ZIKV is malaise, characterized as a pervasive illness, discomfort, or general impression of being unwell.

Regular RT-PCR and quantitative RT-PCR are provided with a quick, precise, and sensitive approach for the early detection of ZIKV [37]. Additionally, diagnostic enzyme-linked immunosorbent assay (ELISA) can be used to find immunoglobulin M (IgM) antibodies to the ZIKV virus. People don't usually get sick enough to be hospitalized and rarely die from ZIKV. For this reason, many people may not realize they have been infected. Symptoms of ZIKV are similar to those of other mosquito-borne viruses, such as dengue and chikungunya. In vitro and in vivo evaluations of the efficiency of metformin (MET) treatment during ZIKV infection were compared to that of MET treatment during DENV infection [38]. ZIKV is pathogenic since it has been linked to severe neurological problems in fetuses, newborns, and adults [39]. Analysis of the included studies is depicted in Table 3.

**Table 3:** Analysis of the included studies

Author	Year	Main characteristics
Musso et al. [1]	2016	Main Characteristics of Zika Virus: in-depth exploration of various aspects of the Zika virus in a comprehensive review.
Plourde et al. [2]	2016	A Review of Zika Virus in Literature: summarizing existing literature on Zika virus in a comprehensive manner.
Pielnaa et al. [3]	2020	Spread, Epidemiology, Genome, and More: overview of Zika virus characteristics, including spread, epidemiology, genome, transmission cycle, clinical manifestation, associated challenges, and progress in vaccine and antiviral drug development.
Sirohi et al. [4]	2017	Zika Virus Structure, Maturation, and Receptors: focus on the structure and receptors of the Zika virus.
Morabito et al. [5]	2017	Development of Zika Virus Vaccines: providing an overview of the progress in developing vaccines for Zika virus.
Christian et al. [6]	2019	Pathophysiology and Mechanisms of Zika Virus Infection in the Nervous System: examining the infection of Zika virus in the nervous system.
White et al. [7]	2016	Zika Virus as an Emerging Neuropathological Agent: exploration of Zika virus as a potential neuropathological agent.
Bhardwaj et al. [8]	2021	Overview of Zika Virus Pathogenesis: summarizing the gist of Zika virus pathogenesis.
Duong et al. [9]	2017	Zika Virus Presence in Asia: discussion on the occurrence of Zika virus in Asia.
Rawal et al. [10]	2016	Zika Virus: A General Overview: providing a general overview of Zika virus.
Duffy et al. [11]	2009	Zika Virus Outbreak in Micronesia: reporting on the Zika virus outbreak in Yap Island, Federated States of Micronesia.
Dupont-Rouzeyrol et al. [12]	2015	Co-Infection with Zika and Dengue Viruses in New Caledonia: a case study on individuals with co-infection of Zika and dengue viruses in 2014.
Wang et al. [13]	2016	Zika Virus and Zika Fever: examining Zika virus and its associated fever.
Yadav et al. [14]	2016	Zika Virus: A Pandemic in Progress: discussing the ongoing global progress of the Zika virus pandemic.
World Health Organization [15]	2023	Information on Zika Virus from the World Health Organization: providing details on Zika virus from the World Health Organization.
Lee et al. [16]	2018	Zika Virus: Transition from Obscurity to Priority: exploring the transformation of Zika virus from obscurity to a global priority.
Foy et al. [17]	2011	Investigation of Non-vector-Borne Zika Virus Transmission in Colorado, USA: exploring the potential non-vector transmission of Zika virus in Colorado, USA.
Turpin et al. [18]	2022	Study on Apoptosis During Zika Virus Infection: examining apoptosis during Zika virus infection and its timing.
Bhardwaj et al. [19]	2017	Current Concerns Related to Zika Virus in India: discussion on the prevailing concerns regarding Zika virus in India.
Rossi et al. [20]	2018	Exploration of Zika Virus Mutations and Severe Outbreaks: investigating whether Zika virus mutations contribute to severe outbreaks.
McArthur et al. [21]	2017	Recent Advances in Zika Virus Vaccine and Therapeutics: providing an update on recent developments in Zika virus vaccine and therapeutic development.
Medin et al. [22]	2017	Zika Virus: Its Biology and Relevance to Pathology: offering an overview of Zika virus and its biological aspects with relevance to pathology.
Cao-Lormeau et al. [23]	2014	Zika Virus in French Polynesia in 2013: reporting on Zika virus in French Polynesia in 2013.
Liu et al. [24]	2017	Enhancement of Zika Virus Infectivity in *Aedes aegypti* Mosquitoes: studying the increased infectivity of Zika virus in *Aedes aegypti* mosquitoes.
Gubler et al. [25]	2017	Historical Perspective on the Emergence of Zika Virus: providing a historical view on the emergence of Zika virus.
Gregory et al. [26]	2017	Examination of Different Modes of Zika Virus Transmission: investigating various modes of Zika virus transmission.
Weaver et al. [27]	2016	Overview of Zika Virus History, Emergence, Biology, and Control Prospects: summarizing the history, emergence, biology, and prospects for controlling Zika virus.
Göertz et al. [28]	2018	Exploration of Functional RNA During Zika Virus Infection: investigating the role of functional RNA during Zika virus infection.
Munoz-Jordan et al. [29]	2017	Challenges and Opportunities in Diagnosing Zika Virus Infections: discussing the difficulties and possibilities in diagnosing Zika virus infections.
Gourinat et al. [30]	2015	Detection of Zika Virus in Urine Samples: identifying Zika virus in samples of urine.
Valerio Sallent et al. [31]	2016	Implications of Zika Virus Infection for the Future of Infectious Diseases: discussing the implications of Zika virus infection for the future of infectious diseases.
Abrams et al. [32]	2017	Approaches to Treating Zika Virus Infection in the Nervous System: discussing various approaches to treating Zika virus infection in the nervous system.
Landry et al. [33]	2017	Laboratory Methods for Diagnosing Zika Virus Infection: describing laboratory techniques for diagnosing Zika virus infection.
Saiz et al. [34]	2017	Efforts in Finding Antivirals for Zika Virus: discussing ongoing efforts to discover antiviral treatments for Zika virus.
CDC [35]	2014	Symptoms Associated with Zika Virus Infection: describing symptoms linked to Zika virus infection.
Yadav et al. [36]	2016	Zika Virus as a New Arbovirus: examining Zika virus as a newly emerged arbovirus.
MedlinePlus et al. [37]	2023	Information on Zika Virus Testing from MedlinePlus: providing details on Zika virus testing from MedlinePlus.
Farfan-Morales et al. [38]	2021	Investigating the Antiviral Effect of Metformin on Zika and Dengue Virus Infections: studying the potential antiviral effect of metformin on Zika and dengue virus infections.
Ferraris et al. [39]	2019	Update on Zika Virus Infection: covering recent developments and information on Zika virus infection.

## Conclusions

This comprehensive narrative study offers a thorough summary of the ZIKV, covering everything from its historical emergence in 1947 to the most recent advancements in 2019. Important topics covered in the review are the virus's genetic makeup, its transmission mechanisms, clinical manifestations, and diagnostic techniques, and the effects of epidemics on a worldwide scale. To obtain insight into ZIKV's evolutionary patterns, the authors emphasize the need to comprehend the less-recognized early occurrences by concentrating on outbreaks that preceded the 2007 Yap Island spike. The study provides an overview of ZIKV's pathogenicity and effects on public health by synthesizing research on the virus's correlation with severe consequences such as microcephaly and Guillain-Barré syndrome. The investigation of diagnostic methods, such as serological assays and RT-PCR, advances our knowledge of various strategies in many contexts, especially those with limited resources. The addition of research on the molecular makeup of ZIKV, the kinetics of its transmission, and the timing of apoptosis during infection enhances our knowledge of the virus on both a molecular and a cellular level. The complicated epidemiology of ZIKV is highlighted by the means of transmission that have been identified, which include sexual contact, mother to fetus, mosquitoes, blood transfusions, and organ donation. Nevertheless, even with these developments, some unanswered questions still call for more study. Further investigation is necessary to determine the exact mechanisms underlying viral-induced cellular stress and immunological responses, particularly in relation to neurological illnesses. To develop more potent preventative and control measures, it is also important to pay close attention to the variables contributing to the creation of further *Aedes* virus infections and the possibility of genetic modifications. In summary, the study brings together a plethora of information on ZIKV. Still, it also highlights how dynamic infectious disease research is. It calls for ongoing efforts to address new problems and close knowledge gaps to effectively manage public health and plan for outbreaks.
